# Identification of Leukemia Subtypes from Microscopic Images Using Convolutional Neural Network

**DOI:** 10.3390/diagnostics9030104

**Published:** 2019-08-25

**Authors:** Nizar Ahmed, Altug Yigit, Zerrin Isik, Adil Alpkocak

**Affiliations:** Department of Computer Engineering, Dokuz Eylul University, 35160 Izmir, Turkey

**Keywords:** leukemia diagnosis, recognizing leukemia subtypes, multi-class classification, microscopic blood cells images, data augmentation, deep learning, convolutional neural network

## Abstract

Leukemia is a fatal cancer and has two main types: Acute and chronic. Each type has two more subtypes: Lymphoid and myeloid. Hence, in total, there are four subtypes of leukemia. This study proposes a new approach for diagnosis of all subtypes of leukemia from microscopic blood cell images using convolutional neural networks (CNN), which requires a large training data set. Therefore, we also investigated the effects of data augmentation for an increasing number of training samples synthetically. We used two publicly available leukemia data sources: ALL-IDB and ASH Image Bank. Next, we applied seven different image transformation techniques as data augmentation. We designed a CNN architecture capable of recognizing all subtypes of leukemia. Besides, we also explored other well-known machine learning algorithms such as naive Bayes, support vector machine, *k*-nearest neighbor, and decision tree. To evaluate our approach, we set up a set of experiments and used 5-fold cross-validation. The results we obtained from experiments showed that our CNN model performance has 88.25% and 81.74% accuracy, in leukemia versus healthy and multi-class classification of all subtypes, respectively. Finally, we also showed that the CNN model has a better performance than other well-known machine learning algorithms.

## 1. Introduction

**Figure 1:**
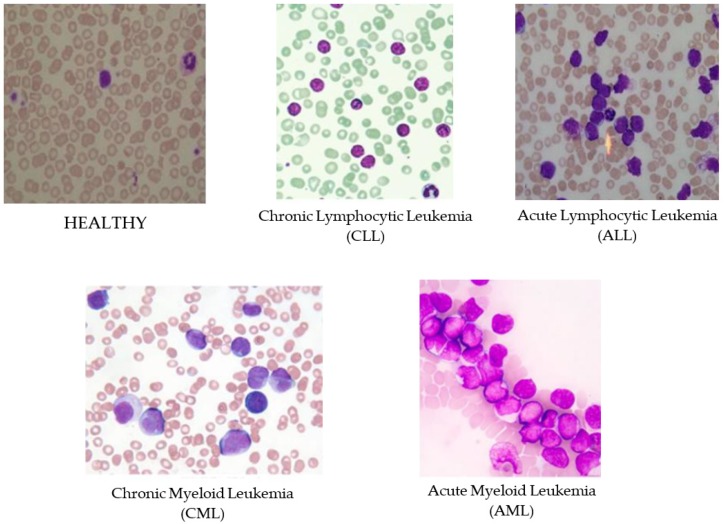
Sample images of four different types of Leukemia: Chronic Lymphocytic Leukemia (CLL) [8], Chronic Myeloid Leukemia (CML) [8], Acute Lymphocytic Leukemia (ALL) [8], Acute Myeloid Leukemia (AML) [8], and HEALTHY [5].

Leukemia is an aggressive disease related to the white blood cells (WBC) and affects the bone marrow and blood of the human body. This disease can lead to destroying the immune system of the human body. There are two main types of leukemia, acute and chronic leukemia, which is depending on how fast it progresses. In acute leukemia, infected WBC do not perform or act like the normal WBC; while it can act as normal WBC in chronic leukemia. Therefore, chronic leukemia can be severe since it cannot be differentiated from normal WBC. Moreover, there are two subtypes of each leukemia types depending on the size and the shape of the WBC: Lymphoid and myeloid. In general, there are four subtypes of leukemia as shown in Figure 1, Acute Lymphocytic Leukemia (ALL), Acute Myeloid Leukemia (AML), Chronic Lymphocytic Leukemia (CLL), and Chronic Myeloid Leukemia (CML) [1,2]. Identifying the existence of leukemia as well as their specific types is important for hematologists to avoid medical risks and specify the right therapy. Thus, using intelligent ways for diagnosis will facilitate and speed up the discovery of leukemia subtypes using the blood cells images (i.e., blood smears).

Microscopic blood tests are considered as the main procedure for the diagnosis of leukemia [2]. Analysis of blood smears is the most common way of discovering leukemia, but not the only one technique. Interventional radiology is an alternative technique for the diagnosis of leukemia. However, radiological techniques, such as percutaneous aspiration, biopsy, and catheter drainage, suffer from inheriting limitations of imaging modality sensitivity and resolution of the radio images [3]. Moreover, other techniques, such as Molecular Cytogenetics, Long Distance Inverse Polymerase Chain Reaction (LDI-PCR), and Array-based Comparative Genomic Hybridization (aCGH), need extensive work and time to identify leukemia types [4]. Due to time and cost requirements of these techniques, microscopic blood tests and bone marrow are the most common methods for identification of leukemia subtypes.

A machine learning (ML) algorithm will help to identify the blood cells with leukemia from the HEALTHY cells when a large training set is available. ALL-IDB leukemia image repository [5] is one of the datasets that is used by a number of medical researchers [1,6,7] as a benchmark. Another leukemia dataset is from the American Society of Hematology (ASH) and it is online at website [8]. Thanh et al. [7] used the ASH database for identifying AML leukemia in their research. Google is another source of leukemia image without annotations, where images were collected randomly from websites. Karthikeyan et al. [9] used the microscopic images collected from Google in their research for identifying leukemia, where authors annotated images by themselves. Successful implementation of machine learning based leukemia diagnosis can be built upon the use of an annotated image dataset.

Identification of leukemia subtypes from the HEALTHY samples is quite a challenging issue. In the literature, many researchers studied only binary classification between one subtype versus HEALTHY samples [1,7,9,10,11,12]. They obtained quite a high performance even more than 96% accuracy. Besides, Shafique et al. [6] further classified samples with ALL subtypes, according to the size of the cell and the nature of its nucleus. However, dealing with the identification of all subtypes of leukemia is more challenging task than simple binary classification [13]. To the best of our knowledge, there is no automatic recognition approach dealing with all subtypes of leukemia. 

Several ML algorithms help to classify and recognize leukemia disease from microscopic images. For example, Paswan et al. [10] who used support vector machine (SVM) and k-nearest neighbor (k-NN) to classify AML leukemia subtype, obtained 83% accuracy. Patel et al. [1] applied SVM for classifying ALL leukemia subtype and achieved 93% accuracy. Karthikeyan et al. [9] also used SVM and c-mean clustering technique to separate WBC from the background and they reached 90% accuracy. Although using a deep learning (DL) approach seems more effective, its performance highly depends on the quantity and quality of dataset used [6]. Convolutional Neural Network (CNN) is one of the neural networks architecture that is generally used to deal with image classification and registration problems. Shafique et al. [6] applied a convolutional neural network (CNN) for identifying ALL leukemia subtypes. Their results recorded 99% for binary classification, between ALL versus HEALTHY samples, and 96% for the further classification of subtypes of ALL only. Thanh et al. [7] also built a CNN model that consists of five convolutional layers to make a binary classification of ALL leukemia subtypes and obtained 96.6% accuracy. Unfortunately, the performance classification in this kind of neural network needs a large training data to learn how to identify important objects from the whole image. However, developing a large training dataset is very time consuming and is a very labor-intensive task. To avoid this problem, we suggest expanding the limited number of samples by image augmentation. Using an inadequate number of image samples in the training dataset may lead to an overfitting problem [14]. Hence, most of the researchers in the literature depend on applying some image transformation techniques to increase the number of training set samples synthetically to avoid an overfitting problem. Patel et al. [1] applied median and Wiener filters to remove noise and blurriness. In the literature, many of the image transformation techniques have been used such as image rotation and mirroring, histogram equalization, image translation, gray scale transformation, blurring images, and image shearing [6,9,10]. Using image augmentation makes it possible to use a DL approach, which requires a large number in the training dataset.

In this study, we propose a new approach for leukemia diagnosis from microscopic blood images identifying the four subtypes of leukemia (i.e., ALL, AML, CLL, and CML) by using CNN architecture of deep learning. To the best of our knowledge, this is the first study dealing with all four leukemia subtypes. Furthermore, the deep neural network requires large training datasets, which is not a trivial problem in our case. Thus, we used an image augmentation technique to increase the number of samples. Additionally, we compared our approach with well-known ML algorithms and evaluated their performances with 5-fold cross validation.

The rest of the paper is organized as follows: Section 2 represents the dataset we used and our method in detail. Section 3 presents the experimentation setups. Results and discussions are given in Section 4. Finally, Section 5 concludes the paper and provides a look at further studies on this topic.

## 2. Materials and Methods

This section presents the details of dataset collection we used, then data augmentation using image transformations and finally applying CNN to classify the four leukemia subtypes.

### 2.1. Dataset

In this study, we collected data from two different sources: ALL-IDB [5] and The American Society of Hematology (ASH) Image Bank [8]. ALL-IDB dataset provided annotated microscopic blood cell images designed for evaluation of segmentation and classification purposes. It included only ALL type of leukemia and HEALTHY samples. ALL-IDB was considered more reliable since, for each image in the dataset, expert oncologists provided the classification/position of ALL lymphoblasts. The other subtypes of Leukemia (i.e., AML, CML, and CLL) did not exist in this dataset.

ASH Image Bank is publicly available on the Web and includes a comprehensive collection of images related to a wide range of hematological topics. In this study, we selected all available blood cell images annotated with leukemia, including any of the four subtypes. Table 1 depicts the number of samples for each of the subtypes and datasets.

### 2.2. Data Augmentation

Data augmentation techniques were widely utilized to increase the dataset size and avoid memorization, especially in deep learning algorithms. Several image transformation techniques, such as shifting, rotation, and flipping, were employed to obtain different versions of original images. When ML models were trained not only with the original image but also with different image versions, they would have more generalization capabilities. Different studies on image classification with CNNs, various data augmentation techniques reduced the error rate of the model by providing better generalization [15,16,17,18,19,20]. 

**Figure 2:**
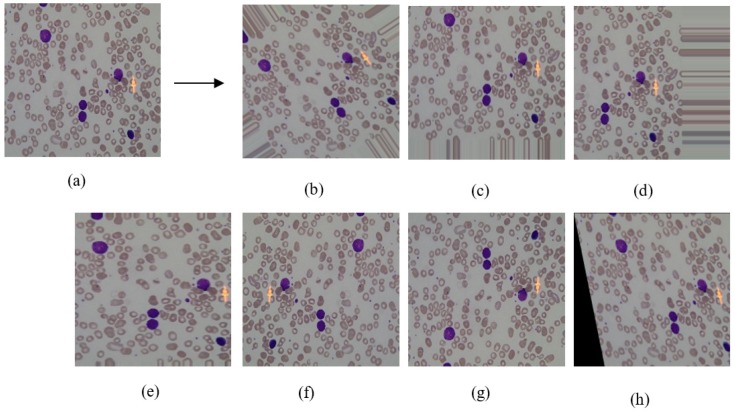
The effect of applying image transformation on one image sample. (**a**) Original image, (**b**) rotation, (**c**) height shift, (**d**) width shift, (**e**) zoom, (**f**) horizontal flip, (**g**) vertical flip, (**h**) shearing.

In this study, utilized datasets provided a good collection of microscopic blood cell images, however, both contained very limited number (i.e., a couple of hundreds) of samples for each leukemia subtype. The exact number of samples for each subtype of leukemia are shown in Table 1. The number of samples was 354 and 549 in the original ALL-IDB and ASH Image Bank datasets, respectively. Data sample sizes were quite few for deep learning methods, hence it could lead to memorization of the algorithm. Therefore, we increased the number of samples using the following seven image transformations [21,22]:

1. *Rotation (40°)*: It was done by giving the rotating effect to the image in a random direction (left or right). During this process, pixel values of an image are moved left, right, up, down according to the degree value specified between 0–180 as shown in Figure 2b. In this study, the degree value was selected at 40° to obtain various images.
2. *Height Shift (40%)*: It was obtained by randomly shifting the image pixels to up or down with 40% as shown in Figure 2c. With the help of this transformation, machine learning models avoid memorizing images that were constantly centered in the dataset.
3. *Width Shift (40%)*: When this operation was performed, the pixel values were moved to the right or left with 40%. After height or width shift operations, a gap occurred in the opposite direction of the shifted direction. It is preferred to fill this gap with neighboring pixels as shown in Figure 2d.
4. *Zoom (30%)*: It makes the appearance of objects in the image closer. It was done by adding new pixel values to the original image. When adding the values, the original pixel values were examined and the nearest value was determined. As a result, the zoomed image was obtained as in Figure 2e.
5. *Horizontal Flip*: In this operation, image pixels were moved horizontally from one half of the image to the other half, as seen in Figure 2f. It was determined that the pixel values would move in a random direction.
6. *Vertical Flip*: The image was divided by a line drawn horizontally from the center of the image. As seen in Figure 2g, pixels were moved vertically unlike horizontal flip.
7. *Shearing (20°)*: It was done by shifting the image pixels counter-clockwise according to the specified angle in degree. In this study, this value set at 20° and the example image is shown in Figure 2h.

We used Python programming language with two open source image processing libraries, OpenCV and KERAS to perform image transformations. Figure 2 shows the applied transformations effect on a sample image. In Figure 2, seven images, which were generated from sample image (a) were shown for each transformation method. After applying image transformation methods, the number of samples increased to 8 times for both data sets as shown in the augmented row values of Table 1. When generating different variations of original images, we applied 7 image transformation methods randomly. After performing data augmentation, the total number of samples reached 2478 and 3843 for ALL-IDB and ASH Image Bank datasets, respectively.

### 2.3. Convolutional Neural Network Architecture

**Figure 3:**
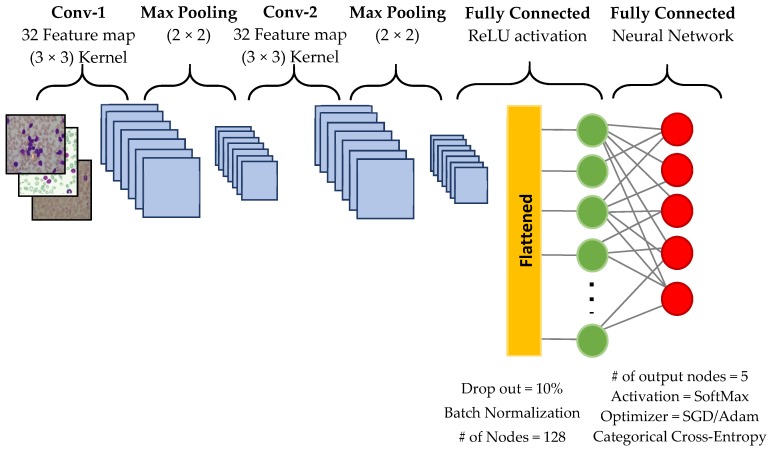
The proposed convolutional neural network (CNN) architecture.

The CNN architecture mainly consisted of convolution layers, pooling layers, flattening, and multilayer perceptron [15,23]. CNNs performed automatic feature extraction from the input images and then classified them with fully connected neural networks. Feature extraction was performed by convolution and pooling layers. After applying filters on the image in these layers, the features were obtained and the classification stage was started. Figure 3 shows the details of the architecture we designed. The details of each phase are described below:

- *Convolutional Layer:* It was responsible for applying several feature detectors to explore many filters on the input image. The CNN we used had a 32 feature map with the size of 3 × 3. Convolution filters were applied to the image by sliding. The filter values were determined randomly. We used 2 convolution layers to avoid overfitting.
- *Max-Pooling Layer:* This layer was responsible for decreasing the dimension of the filtered image thus that it focused on the important feature/area or object in the image. In our network, we used a max-pooling layer with the size of 2 × 2. We doubled the number of this layer, as well.
- *Flatten Layer:* This layer transformed a 2-dimensional max-pooled matrix into one dimensional array thus that each cell of this array could be used as an input node for the full connected network.
- *Fully Connected Network:* This part was a naive connected full forward network that consisted of one input layer (the flattened layer in our case), a hidden layer, and an output layer. In our model, the hidden layer consisted of 128 nodes with 10% Dropout and Batch-Normalization for minimizing overfitting. Due to a simple calculation, the ReLU activation function had been implemented. In the output layer, we setup two types of optimizers SGD (stochastic gradient descent) and ADAM optimizers one type at a time. We added 5 output nodes (each node represents each leukemia type and HEALTHY samples) and all of them were controlled by a SoftMax activation function.

Our CNN model was trained with 25 epochs and 32 batch size since this setup was more suitable with the sample amount of dataset we used. Various numbers of epochs were experimented to obtain the best performance results. We tried to increase the number of epochs to 100, however, it took more running time without significant progress in accuracy.

## 3. Experimentation

We created an experimental environment convenient with the designed CNN architecture. We ran all the experiments in a computer with Core i7 processor and 8 GB RAM running under Windows 10 operating system, and Anaconda 3 with Spider 3.3 and Python 3.6. After setting up the environment, we conducted 4 experiments as follows:

- *Experiment #1*: The primary experiment conducts measuring the first capabilities of the CNN. It was a binary classification experiment without applying image transformations. ALL-IDB dataset consisted of the original ALL subtype and HEALTHY samples with 144 training and 35 test samples for each class. We assigned a dropout parameter and tuned it until becoming suitable with 10% value. In addition, we applied a batch-normalization, which was responsible for minimizing the amount of fluctuations in performance.
- *Experiment #2*: In this experiment, we considered making a binary classification with the same 2 classes in Experiment #1, where image transformation techniques were applied to ALL and HEALTHY samples. Accordingly, we used 980 samples as training data and 245 samples as test data for both classes. Besides, we applied 5-fold cross validation in this experiment.
- *Experiment #3*: This experiment involved the 4 leukemia subtypes: ALL, AML, CLL, CML, and the HEALTHY samples, thus 5 classes were conducted with this experiment. Each leukemia type consisted of 924 training and 231 testing samples. Data augmentation may cause fluctuations in performance. Therefore, we applied the same dropout and batch-normalization parameters as in Experiment #1. Additionally, we considered different epochs numbers: 25 and 100, to observe the effects of a longer time of training. This experiment included the following parameters:
  (a) The Stochastic Gradient Descent (SGD) optimizer with 25 epochs.
  (b) The Stochastic Gradient Descent (SGD) optimizer with 100 epochs.
  (c) The ADAM optimizer with 25 epochs.

We used 2 optimizers to observe which one could decrease the fluctuations in the validation. This may be a result of the irregularity/random selection of the network weights.

- *Experiment #4*: We repeated Experiment #2 and Experiment #3 by using different ML algorithms. This aimed to compare the CNN results with other ML algorithms. We applied this experiment using 4 well-known classifiers: Support Vector Machine (SVM), Naïve Bayes (NB), *k*-NN (with *k* = 3), and Decision Tree (DT). We used 5-fold cross validation for all experiments.

### Evaluation Process

To evaluate our model, we selected 2 main evaluation metrics that were used to measure the performance of any neural network [24,25]. First, *accuracy* was the number of correctly classified image samples among the total number of samples. For neural networks, there were 2 types of accuracy: Training accuracy (TRN-ACC) that measures how good your model in the training process, and validation accuracy (VAL-ACC), which shows how good your model is in classifying unseen data. Secondly, the *loss* is the metric that focuses on calculating the error of prediction, which is used to adjust the weights of neural network nodes. This metric is also calculated in the training process (TRN-LOSS) and validation process (VAL-LOSS).

We divided the dataset into two parts, 70% for training and 30% for testing, before applying any image transformation techniques. Then we applied the image transformations for both parts to increase the number of data samples. We ensured that each part of dataset contained the same number of images with different folds in each cross-validation iteration. Accordingly, we applied our experiments for each fold individually, then we calculated accuracy and loss metrics for each fold. We reported the final performance as an average of 5 folds. 

## 4. Results and Discussion

Table 2 shows the general summary of the results we obtained from each experiment. We obtained the best performance in the binary classification of ALL and HEALTHY classes using augmented samples. The next best score is the multi-classification of the four leukemia subtypes. However, binary classification outperforms the multi-classification problem, since the more classes adhered and involved in a classification process the more complexity will be recognized by the model to differentiate between the classes. We observed that SGD optimizer works much better than ADAM optimizer in terms of both accuracy and loss metrics. Besides, we also observed that longer epoch iterations do not contribute to the performance of the model since *Exp#3a* produced a better result than *Exp#3b*. 

Table 3 and Table 4 present the results of each fold’s performance for binary and multi-classification, respectively. In binary classification, the best score was achieved in fold 4. In the multi-classification, the best performance was obtained in fold 3 in terms of validation accuracy and loss. We can conclude that the selection of samples for test and training set may fluctuate the performance results significantly. Multiple fold cross validation leads to more reliable results in evaluation, thus that we evaluated our approach using 5-fold cross-validation. 

As known, a deep neural network requires large training datasets, since small training set may cause overfitting problem. To overcome this issue, we used data augmentation. The more training samples, the more CNN capability of discovering the patterns which each leukemia type may hold. Although data augmentation plays an important role to increase the sample amounts and to avoid overfitting, they might add some extra noise to the original data. This requires fine-tuning of dropout and batch-normalization parameters; nevertheless, it may not be able to solve problem itself. Besides, it could happen due to very similar structure of blood cells. Performance of multi-classification dropped into 81.74%. This result explains the performance drop and how adding noise with data augmentation degrades the identification of patterns of different leukemia subtypes.

### A Comparison with Other Studies and Methods

We performed a general comparison between our model and the other studies in the literature. Additionally, we compared commonly-used machine learning algorithms SVM, NB, DT, and *k*-NN (*k* = 3) algorithms for the binary and multi-classification as explained in Exp#4. We compared different studies Shafique et al. [6] and Thanh et al. [7] in terms of average accuracy, using ALL-DB dataset for the binary classification of leukemia samples.

Table 5 shows a comparison of the binary classification between ALL and HEALTHY samples for two literature studies versus our proposals, in terms of average accuracy. Our model in CNN has the third best score, which is 88.25% in accuracy. Studies by Shafique et al. [6] and Thanh et al. [7] outperformed our CNN model in the binary classification problem. However, it should be taken into consideration that they did not use any cross validation in evaluation, it is unknown how test and training sets were selected. When we considered the best individual folds as the final score, the CNN model may be considered to achieve 99.3% accuracy, in fold 3. This result is better than reference [7] who obtained 96.6% accuracy for the binary classification of leukemia. 

The results of several models are given in Table 6 for the multi-classification problem. Our model obtained the second-best score with 81.74% average accuracy after the study of Shafique et al. [6] who made a multi-classification of only ALL subtypes, not all subtypes of leukemia. To the best of our knowledge, our study is the first one which explores multi-classification of the four leukemia subtypes. According to Table 4, we get 98.47% accuracy with fold 4, which ranks our model in the top of the multi-classification results. 

**Figure 4:**
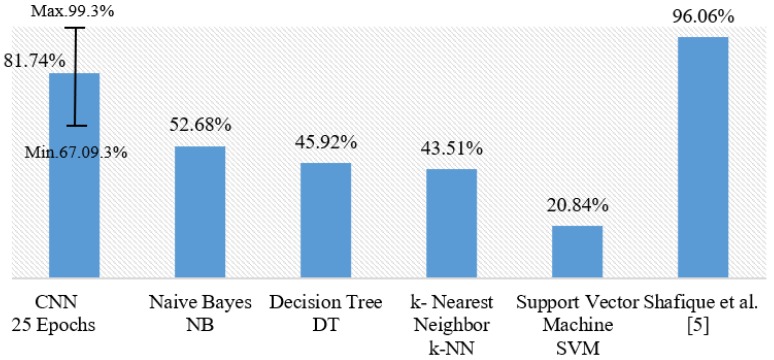
Comparison of our proposals with different machine learning algorithms for the multi-classification of all leukemia subtypes.

Furthermore, our model using SGD optimizer generally outperforms all classical ML classifiers especially NB, which provided the highest score in comparison with DT, SVM, and *k*-NN. Moreover, SGD outperforms the ADAM optimizer in our CNN model for the multi-classification. We also noticed that working with 25 epochs performs better than longer epoch periods. Figure 4 shows a general comparison of all the multi-classification models discussed in this section. Our model with CNN produced 81.74% for multi-classification on average with 5-fold cross validation. The error bar in Figure 4 indicates the minimum and the maximum values of observations in terms of accuracy. Our model produces competing results when the best fold has been taken into consideration. 

## 5. Conclusions

Leukemia is an aggressive cancer that affects the white blood cells and bone marrow and weakens the immune system of the human body. One of the most commonly used diagnosis is based on microscopic blood cells (blood smears) analysis. In this study, we present a new approach for leukemia diagnosis from microscopic blood images by using a CNN architecture, which is capable of identifying the four subtypes of leukemia. Our model proved its competency to deal with the limited number of image samples by applying data augmentation techniques helping to solve the overfitting problem. As a result, we also showed that our CNN outperforms the other machine learning algorithms by achieving 88% accuracy for the binary classification of one leukemia type (ALL and the HEALTHY samples), and an 81% accuracy for classifying all leukemia subtypes. Besides, we did apply cross-validation in all experiments. It is indeed so expensive in execution time for medical image classification, but it is important to see if the model in hand is stable amongst the entire classification process. 

In future work, we plan to expand our experiments by using a hybrid deep learning approach using convolutional neural network accompanied by recurrent neural networks to enhance the performance. Furthermore, we plan to enlarge our dataset by adding new samples as well as using new data augmentation techniques.

## Figures and Tables

**Table 1:** Dataset coverage for four leukemia subtypes and HEALTHY image samples. The original rows indicate the initial sample numbers, the augmented rows show the increased number of samples by using data augmentation.

Dataset	Type	Acute		Chronic		HEALTHY	Total
		Myeloid	Lymphoid	Myeloid	Lymphoid		
ALL-IDB	Original	-	179	-	-	175	354
ALL-IDB	Augmented	-	1253	-	-	1225	2478
ASH Image Bank	Original	179	-	185	185	-	549
ASH Image Bank	Augmented	1253	-	1295	1295	-	3843

**Table 2:** Accuracy and loss results for each experiment.

Exp ID	Experiment Description	Accuracy Type		Loss Type	
		TRN-ACC	VAL-ACC	TRN-LOSS	VAL-LOSS
Exp#1	Binary classification ALL and HEALTHY	99.55%	81.16%	0.0149	1.3093
Exp#2	Binary classification ALL and HEALTHY	99.90%	88.25%	0.0033	0.5653
Exp#3a	Multi-classification with SGD optimizer	99.34%	81.74%	0.0207	1.1419
Exp#3b	Multi-classification with SGD optimizer	99.77%	66.41%	0.0077	2.3665
Exp#3c	Multi-classification with ADAM optimizer	99.36%	63.40%	0.0203	2.6636

**Table 3:** Accuracy and loss scores for the binary-classification experiment (Exp#2) with the detailed results of each fold of the cross validation.

Metrics	Fold 1	Fold 2	Fold 3	Fold 4	Fold 5	Average
VAL-ACC	65.16%	96.32%	90.66%	98.47%	90.66%	88.254%
TRN-ACC	99.87%	99.89%	99.91%	99.92%	99.91%	99.9%
VAL-LOSS	1.9519	0.0997	0.3711	0.0325	0.3711	0.56526
TRN-LOSS	0.0044	0.0036	0.003	0.0026	0.003	0.00332

**Table 4:** Accuracy and loss scores for the multi-classification experiment (Exp#3a) with the detailed results of each fold of the 5-fold cross validation.

Metrics	Fold 1	Fold 2	Fold 3	Fold 4	Fold 5	Average
VAL-ACC	76.17%	94.74%	99.30%	71.37%	67.09%	81.74%
TRN-ACC	99.62%	98.49%	99.19%	99.68%	99.76%	99.35%
VAL-LOSS	1.0058	0.1995	0.0207	2.426	2.057	1.1419
TRN-LOSS	0.0120	0.0468	0.0268	0.009	0.008	0.0207

**Table 5:** A comparison of the binary classification for two literature studies versus our proposal, in terms of average accuracy.

Model		Dataset Used	Accuracy
Shafique et al. [6]		ALL-DB	99.50%
Thanh et al. [7]		ALL-DB	96.60%
Our Proposal	CNN	ALL-DB	88.25%
	NB	ALL-DB	69.69%
	DT	ALL-DB	62.94%
	3-NN	ALL-DB	58.57%
	SVM	ALL-DB	50.09%

**Table 6:** A comparison of the multi-classification for literature studies versus our proposal in terms of average accuracy.

Model		Dataset Used	Accuracy
Shafique et al. [6]		ALL-DB	96.06%
Our proposal	CNN - 25 epochs	ALL-DB, ASH Image Bank	81.74%
	CNN - 100 epochs	ALL-DB, ASH Image Bank	66.41%
	NB	ALL-DB, ASH Image Bank	52.68%
	DT	ALL-DB, ASH Image Bank	45.92%
	3-NN	ALL-DB, ASH Image Bank	43.51%
	SVM	ALL-DB, ASH Image Bank	20.84%
